# Meteorological Data Analysis Using MapReduce

**DOI:** 10.1155/2014/646497

**Published:** 2014-02-23

**Authors:** Wei Fang, V. S. Sheng, XueZhi Wen, Wubin Pan

**Affiliations:** ^1^Jiangsu Engineering Center of Network Monitoring, Nanjing University of Information Science & Technology, Nanjing 210044, China; ^2^School of Computer & Software, Nanjing University of Information Science & Technology, Nanjing 210044, China; ^3^Computer Science Department, University of Central Arkansas, Conway, AR 72035, USA

## Abstract

In the atmospheric science, the scale of meteorological data is massive and growing rapidly. *K*-means is a fast and available cluster algorithm which has been used in many fields. However, for the large-scale meteorological data, the traditional *K*-means algorithm is not capable enough to satisfy the actual application needs efficiently. This paper proposes an improved *MK*-means algorithm (*MK*-means) based on MapReduce according to characteristics of large meteorological datasets. The experimental results show that *MK*-means has more computing ability and scalability.

## 1. Introduction

In the atmospheric sciences, meteorological data is extremely rich and valued, which requires a mass of scientific computing, and provides services to the communities. With the further expansion of meteorological services and the improvement of the modernization standard in meteorology, a large amount of meteorological information has been accumulated and collected in meteorological services, research and management activities. High-performance computers are required to process these data, but small organizations and units cannot afford the high price of high-performance computers. Cloud Computing technology provides the cheap computing services for the Meteorological Organization with higher efficiency, lower cost, and lower carbon. Climate data are dramatically increasing in volume and complexity, since users of these data in the scientific community and the public are rapidly increasing [1]. Faced to such large-scale meteorological data, high-efficient computing power (more than a trillion times) is urgently required. Therefore, establishing a cloud computing weather information processing system is very important and significant.

MapReduce is a key technology of using cloud computing to process a large amount of data. It is a parallel programming model and an associated implementation for processing and generating large datasets in a broad variety of real world tasks proposed by Google. It is not only a programming model, but also a task scheduling model. It is compose of two fundamental functions: Map and Reduce, defined by users. A Map function is to handle a key/value pair to produce intermediate key/value pair. A Reduce function is specified to combine all of the intermediate value with the same middle key [2]. MapReduce is typically used to perform distributed computing on clusters of computers. Google's MapReduce abstracts the distributed computing from its complex details; such that programmers can handle large distributed system resources without any experience about a parallel or distributed system. Thereby, the effect originally achieved only by expensive high-performance computer can be achieved by low-cost computing services.

As we know, not all data mining algorithms can be parallelized to handle large datasets at this moment. Some algorithms cannot be parallelized in theory. Some need to be adapted to take the advantage of the efficiency of parallelization. In this paper, we utilize the *K*-means algorithm in the MapReduce framework. Specifically, we adapt the *K*-means algorithm in an open-source software framework: Hadoop, and apply the parallel *K*-means algorithm (*MK*-means) to cluster the large-scale weather data.

This paper is organized as follows. Related work is reviewed in Section 2. In Section 3, we introduce the MapReduce programming model. In Section 4, we describe our parallel *MK*-means algorithm (*MK*-means) for large-scale meteorological data using MapReduce. In Section 5, we conduct the experiment to evaluate the *MK*-means algorithm by applying it to cluster large-scale meteorological data. Finally, we conclude the paper in Section 6.

## 2. Related Work

In recent years, there is significant research in *K*-means clustering and MapReduce. *K*-means clustering problem has been well studied in data mining research and related fields. *K*-means is one of the top 10 algorithms in data mining [3]. Its simplicity and speed allow it to run on large datasets. With the development of information technology, the volume of information is becoming more and more enlarging. MapReduce is a quite novel programming model for solving certain kinds of distributable problems and processing large datasets [2]. So, to deal with high dimensions and large datasets, some researchers have proposed some methods to solve these problems [4–8]. Böse et al. [9] implemented several incremental data mining algorithms including Naïve Bayes and PCA and applied their methods to deal with large-scale datasets. Chu et al. [10] realized a few algorithms based on MapReduce, such as SVM, ICA, PCA, Gaussian Discriminant Analysis, EM and Backpropagation. Chao et al. [6] proposed a parallel Co*MK*-means algorithm based on MapReduce, which basically distributes the clustering load over a given number of processors. Reference [8] adapts an ensemble learning method-bagging to overcome the instability and sensitivity to outliers in clustering on large datasets. There has been work on developing algorithms and approximation algorithms that fit into the MapReduce [11]. Apache Hadoop [2] is a free Java MapReduce framework that allows the parallel or distributed processing of large datasets. Zhao et al. [4] presented a fast parallel *K*-means clustering algorithm based on the MapReduce framework; however, their approach does not consider the characteristics of large meteorological datasets and cannot achieve good results. Reference [12] demonstrated the utility of the *K*-means clustering algorithm for identifying relationships between winds at turbine heights and climate oscillations, thereby developing a method for predicting the impacts of climate changes on wind resources. However, only a few studies on dealing with the large-scale meteorological data using MapReduce have been reported.

In this paper, we present a parallel clustering algorithm *MK*-means which is based on both *K*-means and MapReduce for very large meteorological data.

## 3. MapReduce Overview 

As said before, MapReduce is developed by Google. Its libraries have been written in many programming languages, such as Java, Python, and C++ [13–16]. It is mainly used to process large-scale (TB-level) data files. MapReduce is not only a simplified programming model, but an efficient distributed scheduling model. Programming is very simple in such a cloud computing environment. The treatment of clusters is handled by the platform, including the reliability and scalability [17]. Application developers only need to focus on the application itself. “Map” and “Reduce” are the two basic computing units of the MapReduce model. Massive data is cut into unrelated blocks by Map program, and scheduled to lots of computers to process, achieving distributed computing. Then the results from these computers are summarized and outputted by Reduce program.

In MapReduce, massive data is processed in parallel. Data is initially partitioned across the nodes (computers) of a cluster and stored in a distributed file system (DFS). Data is represented as (key, value) pairs. The computation of the two functions is expressed formally as follows [5]:

 map(*k*_1_, *v*_1_) → list(*k*_2_, *v*_2_),
 reduce(*k*_2_, list(*v*_2_)) → list(*k*_3_, *v*_3_).

**Figure 1:**
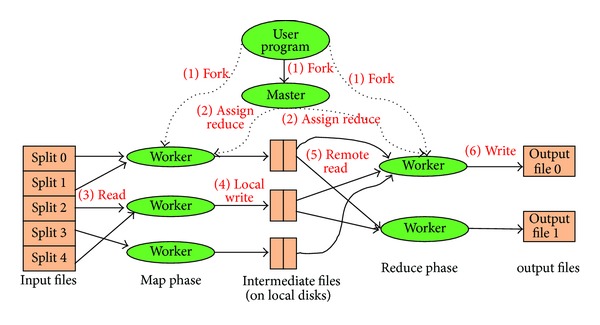
Google's MapReduce programming model.

The Google's MapReduce programming model is shown in Figure 1.

To further understand the MapReduce programming model, the pseudocode of program based on MapReduce is shown in Algorithm 1. The program is used to calculate the annual maximum temperature [18].

A Map function is used to extract all the years and temperatures (key/value pairs) appeared in text, and these pairs are sent to an intermediate temporary space specified by MapReduce. Through intermediate processing by the Map function, the key/value pairs are grouped according to the key, so that each year is followed by a list of temperatures. Then, a Reduce function is only to find the maximum number through a whole list. The result is the annual maximum temperature.

**Figure 2:**
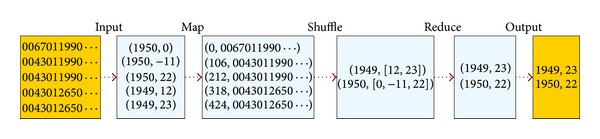
An example of the execution process of MapReduce, including the intermediate results of each step.

Figure 2 shows the intermediate results of each step of the execution process of MapReduce, including Map and Reduce phases, which both use all nodes in the cluster. Between the Map and Reduce phases, there is an intermediate phase, which concatenates the intermediate results with the same key into a list. The list will be used by the Reduce function to output the maximum temperature of a certain year.

## 4. *MK*-Means Clustering Algorithm

*K*-means is a clustering algorithm based on partition. It is widely used in various cluster analyses. This algorithm has good clustering effect in data with spherical, convex distribution, but, for massive datasets, it encounters the bottleneck of efficiency in calculating the distance between objects. It is only guaranteed to converge to local optimum. Its clustering results are very sensitive to the choice of initial centroids. Most importantly, it is not efficient for processing massive data. In this section, we present how to adapt *K*-means in the parallel environment for big data.

Let us briefly review the *K*-means algorithm. Here is the formal description of *K*-means.

Given a set of data points *P* ∈ *R* ^*d*^ and indicated *K* clusters, the goal of *K*-means is to find the *K* centroids *C* = {*c*_1_, *c*_2_, …, *c*_*k*_}, to minimize ∑_*p*∈*P*_[*d*(*p*,*C*)]^2^, where *d*(*p*, *C*) = min⁡_*c*_*i*_∈*C*_{*d*(*p*, *c*_*i*_)}. In order to find the optimal *K* centroids, the *K*-means algorithm initially randomly selects *K* central points in the *d*-dimensional space. Then the *K*-means algorithm calculates the distances of each data point to the *K* centroids, and assigns the data point to the closest centroid. After all data points are assigned to their closest centroid, the initial *K* clusters are formed. For each cluster, *K*-means readjusts its centroid via computing the mean of each dimension of the data points in the cluster. Thus, the *K* centroids are updated. With the updated *K* centroids, *K*-means reassigns all the data points to each centroid again. This process repeats until no more changes of the assignments of all data points.

**Figure 3:**
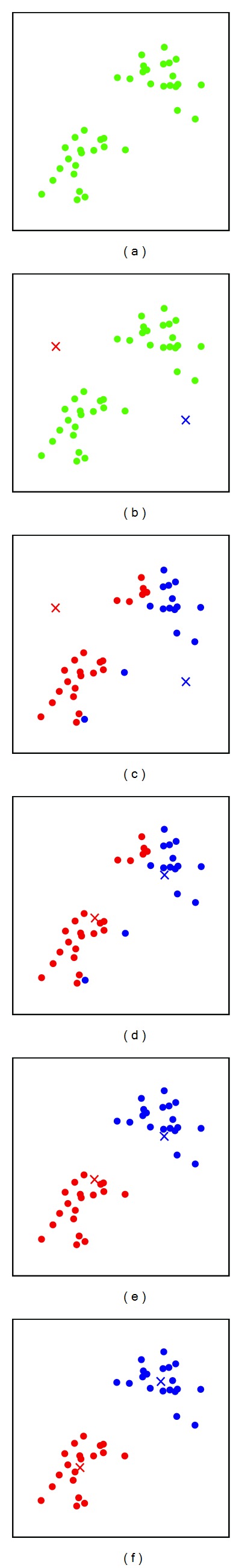
The clustering process of *K*-means.

First, *K* objects chosen from *n* data objects are served as initial cluster centers; for the rest objects, the distances between each central point and all the rest points are not calculated during updating the *K* central point circularly. Instead, the distance between a central point and all points is calculated based on the clustering result, taking the mean. Central points are obtained for the next cycle. The clustering process of *K*-means () is shown in Figure 3. From the figure, we can clearly see that the selected cluster centers are iteratively processed until the final stable status. Then as shown in the red circle, the clustering result is finalized. Thus, the *K* classes obtained by clustering are assigned to each computer node, the central point of *K*-means is calculated by these nodes, and finally the *K* central points are returned. The distance between all data and each central points is calculated to obtain clustering results. The *K*-means algorithm commonly uses Euclidean distance as the standard measure of similarity evaluation. The clustering effect of the objective function *F* can be defined as: For example a specific number such as *K* = 2.

The *K*-means algorithm tries to find an optimal solution by minimizing the square error:

(1) $$\begin{array}{c} \text{Er}=\overset{K}{\underset{i=1}{\sum }}\overset{n}{\underset{j=1}{\sum }}{||x_{j}-c_{i}||}^{2}, \end{array}$$

where *K* is the number of clusters, *n* is the total number of data objects, *c*_*i*_ is the center of the *i*th cluster, and ||*x* − *c*_*i*_|| is the Euclidean distance between the sample *x* and the center *c*_*i*_ of the *i*th cluster.

**Definition 1**

The definition of a set of the centroid points *P* is *c*(*P*) = (∑_*p*∈*P*_ *p*)/|*p*|. Let *C* is a set point of the *d*-dimensional space, 0 < *ε* < 1, if *C*′ meets Δ^2^(*P*, *C*′)≤(1 + *ε*)Δ^2^(*P*); then it is claimed that *C*′ be the *ε* approximate centroid point set of *P*, where Δ_*k*_^2^(*P*) is the optimal value of the cluster results of *K*-means.

**Definition 2**

Let *x*_1_, *x*_2_, …, *x*_*l*_ be the *k* points of *d*-dimensional space, If the existence of *l* real number *u*_1_, *u*_2_, …, *u*_*l*_ to meet: $0\leq {\underline{u}}_{l}\leq 1$  (*i* = 1, 2, …, *l*), ∑_*i*=1_^*l*^ *u*_*i*_ = 1. For a point *x* ∈ *R* ^*d*^ in the *d*-dimensional space, if *x* = *u*_1_ *x*_1_ + *u*_2_ *x*_2_ + ⋯+*u*_*l*_ *x*_*l*_ is established, then *x* is *x*_1_, *x*_2_, …, *x*_*l*_ convex combination of points.

**Theorem 3**

To the fixed-point set *P*, for any a point *x* ∈ *R* ^*d*^,

(2) $$\begin{array}{c} \Delta \left(P,x\right)=\Delta \left(P,c\left(P\right)\right)+\left|P\right|\Delta \left(c\left(P\right),x\right). \end{array}$$

**Figure 4:**
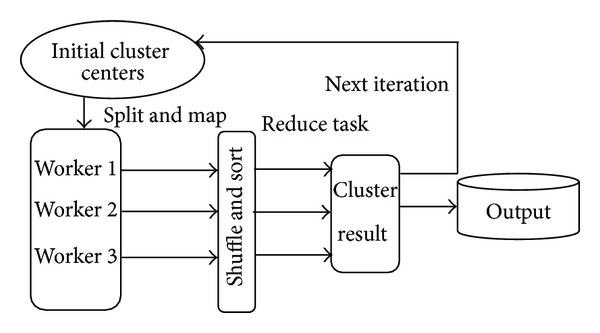
The workflow of the Parallel *K*-means with MapReduce.

In order to take the advantages of high performance parallel computing in meteorological fields, we propose a fast *MK*-means algorithm for weather information processing using the MapReduce model. The parallel workflow of the *MK*-means algorithm is shown in Figure 4.

Figure 4 represents the running process of Parallel *K*-means based on a MapReduce execution. The MapReduce process first splits the data into *N* segments [16]. Then the Map task generates a sequence of 〈key, value〉 pairs from each segment, which are stored in HDFS files. For each Map task, the Parallel *K*-means constructs a global variant center of the clusters. Next the library runs many copies of the program on the Hadoop in a cluster. Then, the intermediate 〈key′/value′〉 pairs are stored in the memory, and are shuffled and sort the 〈key′/value′〉 pairs. Finally, the Reduce function task sums all samples and computes the total number of samples assigned to the same cluster. So, we can obtain the new centers which are used for next iteration.

Then, an *MK*-means clustering algorithm for meteorological data proposed in this paper is shown in Algorithm 2.

*Map Function*. first constructs a global variable list center. Then it scans the sequence file of 〈key, value〉 pairs as an input, and reads each line as a data sample. Then, it calculates the distance of the data samples stored in centers to each centroid, and chooses the minimum distance. The data samples are assigned to the cluster center, and set a value to the data samples. The pseudocode of the Map function is shown in Algorithm 3.

Mapfunction process according to the meteorological services is

 map output 〈key′, value′〉 pair
 Worker 1: (. *A*), (. *B*), (. *C*).
 Worker 2:
 Worker 3:

*Combine Function*. Through each map task, it comes out a large amount of data. In order to reduce the burden of communication among different nodes, the combine-function sums the value of the points assigned to the same cluster with the mean value, then passes the mean value to the reduce function to deal with. The pseudocode for the combine-function is shown in Algorithm 4.

*Reduce Function*. First we obtain the mean value of the combined task from each node, and then combine the local mean value to the global mean value. Input values for Reduce are grouped from intermediate results automatically. To set a counter count in the combine-function, both the Reduce function and the combine-function can contact each other easily. The count also can record the number of data samples involved the mean value. The pseudocode for the Reduce function is shown in Algorithm 5.

## 5. Experimental Classification Results and Analysis

The *MK*-means algorithm is deployed in the meteorological information data center to analyze the meteorological information at the Nanjing University of Information Science & Technology. The meteorological data is described in the following subsection.

### 5.1. Meteorological Dataset

All experiments are conducted on a set of meteorological datasets (http://www.atmosphere.csdb.cn/page/showEntity.vpage?uri=data_ziliao.haiyangziliao). Test data involves 4 years' total factor mapping data of national reference climatological stations from 2004 to 2007 from a China Meteorological Data Sharing Service System (http://cdc.cma.gov.cn/). The data includes monitoring data at 2:00, 8:00, 14:00, and 20:00 every day of 753 national reference climatologically stations all over the whole country. In monitoring data, there are: total cloud amount, wind direction, wind speed, sea level pressure (or the site air pressure), 3 hours transformer, past weather 1, past weather 2, 6 hours of rain, low cloud-like, low cloud cover, low cloud high, dew point, visibility, present weather, temperature, cloud-like, high cloud, and other weather elements the default value of elements is 9999.

The data used in our experiments has 26 attributes: District station number (long integer), longitude, latitude, altitude (both floating-point), site-level (integer), total cloud cover, wind direction, wind speed, sea level pressure (or pressure site), 3 hours transformer, past weather 1, past weather 2, 6 hours of rain, low cloud-like, low cloud cover, low cloud high, dew point, visibility, present weather, temperature, cloud-like, high cloud, flag 1, flag 2 (all integers), and 24 hour variable temperature, 24-hour transformer.

The dataset is a HDFS specified file in Hadoop. We have formed four datasets shown in Table 1. Dataset 1 is the meteorological data of 2007. Dataset 2 is the meteorological data from 2006 to 2007. Dataset 3 is the meteorological data from 2005 to 2007. Dataset 4 is the meteorological data from 2004 to 2007. The properties of the datasets are shown in Table 1. The datasets experimentally selected have the same characteristic, whose class attribute is numerical.

### 5.2. Experiment Platform

The experiment is conducted on nine PCs running an operating system CentOS5.4 (Red Hat Enterprise Linux 4.1.2). Each PC installs the related software, such as jdK-1.6.0, Hadoop-0.19.2 and Mahout-0.3. The distributed cloud environment is based on Hadoop. Its nodes are divided into NameNode and DataNode (only one NameNode and multiple DataNodes). In the view of MapReduce, nodes can be divided into JobTracker and TaskTracker (only one JobTracker and multiple TaskTrackers). JobTracker and NameNode can be deployed on the same machine. The machine deployed NameNode and/or JobTracker is master, the rest are slavers.

**Figure 5:**
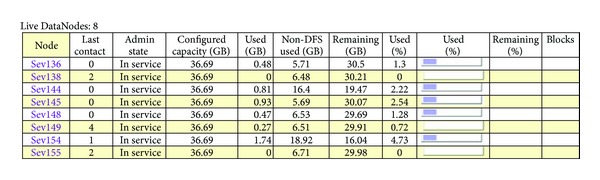
The example of the status of our Hadoop cluster.

In the experiment, nine PCs are used to build the cloud computing environment. Each PC uses the CPU of Intel Core 2.66 GHz, with 2 G RAM. Nine computers are connected through a 100 Mbps LAN switch. We have a label for each PC. “Aiken” is served as NameNode and JobTracker, sev136, sev138, sev144, sev145, sev148, sevl49, sevl54, and sevl55 are served as DataNodes and TaskTrackers. The directory (/etc/hosts) of each machine is configured. The IP of NameNode and JobTracker is configured under the directory conf/hadoop-site.xml. After the Hadoop cluster is built successfully, the information of each node and the information of the MapReduce tasks are shown in Figure 5.

The server “Aiken” can login each machine without password via ssh-keygen. The key configuration items of Hadoop in the experiment environment are shown in Table 2. Each machine modifies conf/masters. Again, the IP of NameNode and jobtracker is configured under conf/hadoop-site.xml. The relevant parameters are modified by conf/hadoop-default.xml, and conf/hadoop-site.xml.

During the experiment, we found an important factor: block size, which impacts the performance significantly. If the block division is set to too small, the job will increase the number of collaboration and increase the cost of reduced performance. Otherwise, it cannot maximize the benefit of parallel processing. So the block size for data processing should based on the amount of the real required size.

### 5.3. Experimental Results

To evaluate the performance of our proposed *MK*-means algorithm for meteorological datasets, we use the running time, speedup, scaleup to validate it [19]. Speedup describes the performance of a parallel algorithm. It is like the reduced running time. As we know, the reduced run time is an important indicator to verify the performance of a parallel algorithm. Speedup *S* is defined as: *S* = *T*_*s*_/*T*_*p*_, where *T*_*s*_ is the time it takes to solve the problem on a single machine, and *T*_*p*_ is the time spent by a parallel algorithm in the same node for solving the same problem. With the increase of *m*, the *MK*-means algorithm can still maintain a linear growth status, then provides more nodes to shorten the time spent.

We first conduct the experiment on the four datasets described in Table 1. The intermediate results of each iteration are stored in the “clusters-*X*” folder, where “*X*” is the number of clusters. The final clustering results are stored in the folder of points. The results are shown in Table 3.

The experimental results show that, the *MK*-means algorithm is suitable for the actual situation. The running procedure of the *MK*-means algorithm is stable and reliable, and the requirements of large data processing in the actual parallel and distributed environment can be satisfied.

Meanwhile, we further investigated the performance of the *MK*-means algorithm with different number of nodes used in Hadoop. In addition, we also investigated the performance of the *MK*-means algorithm with different size of datasets. The corresponding running time is shown in Figure 5.

**Figure 6:**
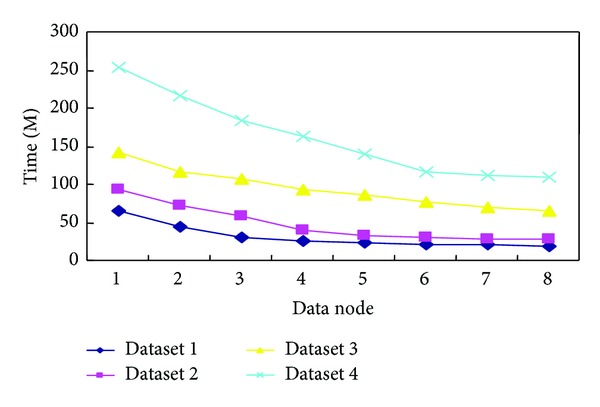
Test results of different datasets.

**Figure 7:**
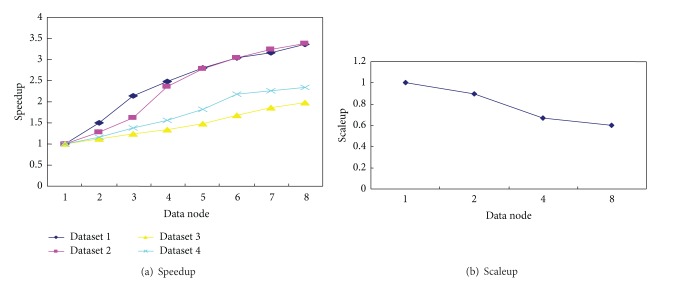
System evaluation results.

Figure 6 shows that the running time of the *MK*-means algorithm decreases with the corresponding increment of the number of nodes used. The dataset size is large, the more significant the running time reduces with the number of computer nodes. We also evaluate the performance of the *MK*-means algorithm in terms of speedup and scaleup, shown in Figure 7. As we described before, speedup also measures the performance of the *MK*-means algorithm. Besides, we also measure the scalability (Scaleup) of our algorithm. Evaluation of scaleup is to increase the number of nodes in expanding the same amount of data at the same time. Scaleup is defined as follows:

(3) $$\begin{array}{c} \text{scaleup}\;\;\left(DB,m\right)=\frac{DB\;\text{calculated}\;\text{in}\;\text{a}\;\text{node}}{m*DB\;\text{calculated}\;\text{in}\;m\;\text{nodes}}. \end{array}$$

If the value of scaleup is in the vicinity of 1, or less, with the change of *m*, it means that the algorithm has very good adaptability on the dataset. The result of scaleup is shown in Figure 7(b).

In our experiment, the number of nodes varies from one to eight; the data size of the dataset increases from 1 G to 10.8 G.  Figure 7(a) shows the speedup values for different number of nodes. It is shown that our algorithm has reasonable speedup performance. On four different size datasets, the speedup of our algorithm consistently goes up when more nodes are available. Then, as the size of the datasets increases, the speedup performs better. Figure 7(b) shows that how well the fast *MK*-means algorithm deals with large datasets when more computer nodes are available. Obviously, the *MK*-means algorithm has very good scalability. This system is deployed in the meteorological information data center to analyze the meteorological information at the Nanjing University of Information Science & Technology. In the real-world situation, it is stable and reliable, and meets the needs of analyzing the large meteorological data.

To validate the *MK*-means algorithm for meteorological data efficiently, we have compared *MK*-means with *PK*-means [4]. The two algorithms, both with the Map-Reduce framework for clustering, are comparable.

**Figure 8:**
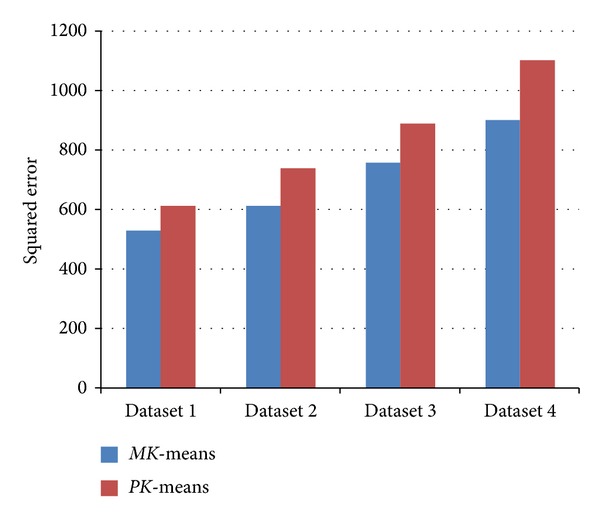
The square error of clustering results.

In addition, the squared-error criterion is used to measure the result of clustering, defined as:

(4) $$\begin{array}{c} E=\overset{K}{\underset{i=1}{\sum }}\overset{n}{\underset{p\in C_{i}}{\sum }}{\left|p-m_{i}\right|}^{2}, \end{array}$$

where *E* is the square error summation for all objects in the dataset, *p* is a given object in cluster *C*_*i*_, and *m*_*i*_ is the mean of cluster *C*_*i*_. The comparative evaluation of the square error between *MK*-means and *PK*-means [4] is shown in Figure 8.

From Figure 8, it is easy to notice that the square error of *MK*-means is significantly lower than that of *PK*-means. It shows that *MK*-means can improve the stability of the *K*-means algorithm for meteorological data, and our *MK*-means can partly solve the problem of the instability and sensitivity to outliers of *K*-means.

We also investigate the impact of the file size. In our experiments, we have two contrast datasets (dataset 5 and dataset 6 shown in Table 4), whose total sizes are the same (230 MB). Dataset 5 has 1217 small files, and its file size is between 250 KB and 500 KB; Dataset 6 has one large file (about 230 MB). Hadoop default data block size is 64 MB. The throughputs of the two different type datasets are shown in Table 4. From Table 4, we can see that the throughput of dataset 5 (with a large number of small files) is much less than dataset 6 (with one large file) in the system. Therefore, we can conclude that Hadoop has the advantage on handling large size files. This is because a lot of time is wasted on the process of reading and writing a large number of small files during the Map operation.

## 6. Conclusion

With the development of cloud computing, research on distributed parallel algorithms attracts more and more attention. There exist some parallel classification and clustering algorithms. However, an effective and cheap solution for processing the massive meteorological information is highly demanded. In this paper, we initiated a meteorological information processing system based on cloud computing and compared with some existing approaches. Then, we proposed a fast *MK*-means clustering algorithm for analyzing meteorological information processing using MapReduce. After having built the Hadoop experimental platform, we investigated the performance of our *MK*-means algorithm. Our experimental results show that our *MK*-means algorithm deployed in the large-scale meteorological data processing system is feasible and efficient. Next, we will further optimize the algorithm and integrate the system with other parallel and distributed algorithms into the system to meet with the challenge of Big Data.

## Figures and Tables

**Algorithm 1:**
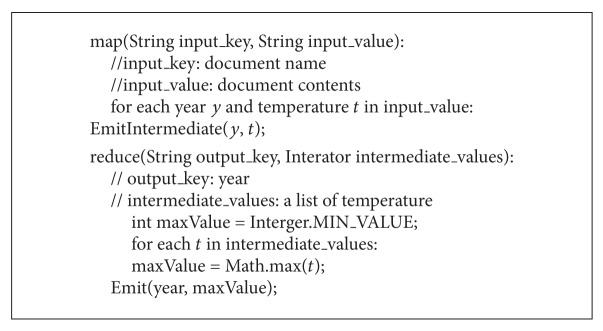


**Algorithm 2:**
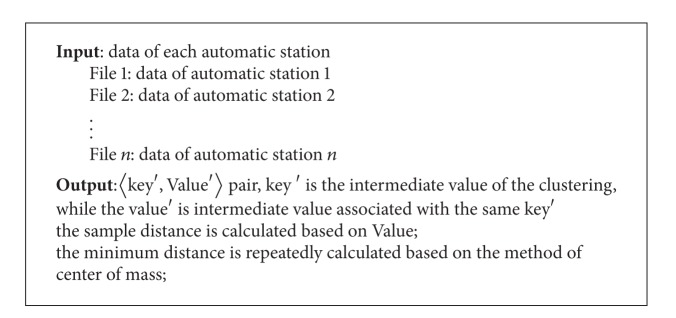


**Algorithm 3:**
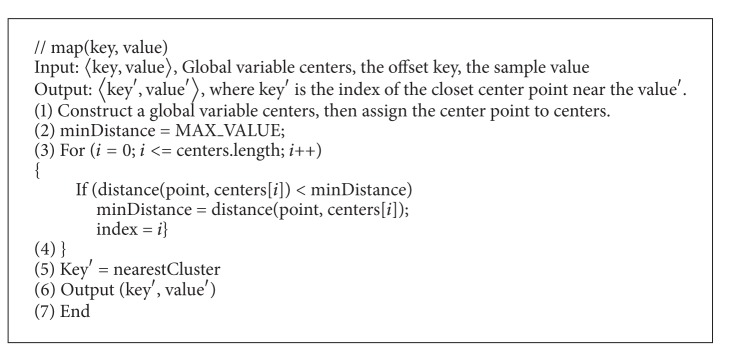


**Algorithm 4:**
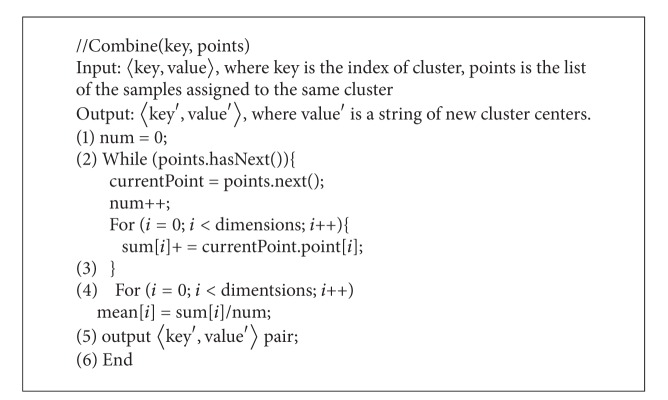


**Algorithm 5:**
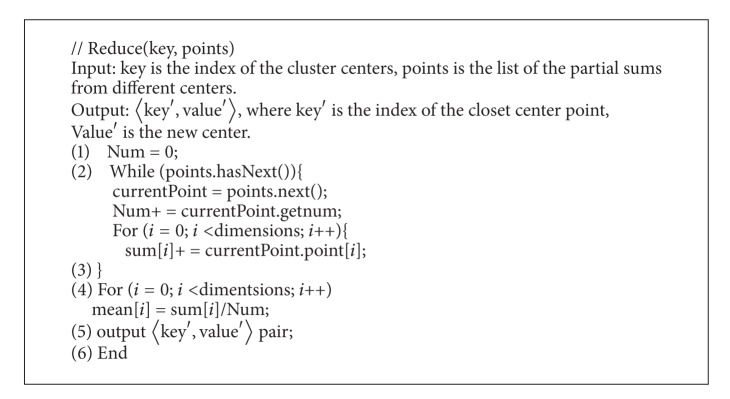


**Table 1:** Experimental datasets.

Dataset	File name	Capacity	Matrix	Type
1	dataset1.txt	250 M	2.5∗106∗26	1 year dataset
2	dataset2.txt	500 M	5∗106∗26	2 years dataset
3	dataset3.txt	1 G	1∗107∗26	3 years dataset
4	dataset4.txt	2 G	2∗107∗26	4 years dataset

**Table 2:** The configuration items of Hadoop key parameters.

Configuration parameter name	Parameter value	Description
io.sort.mb	256	Maximum Memory to store temporary data in the phase of arrangement, overflow to the disk if excess, unit: M
dfs.replication	3	Number of file backup
dfs.block.size	409600	The maximum value of each file: the file is read and stored in block if excess unit: bit
mapred.local.dir	/mapred/local	Data stored path when MapReduce task executes
mapred.tasktracker. map.tasks.maximun	2	The maximum number of Map tasks can be run on a TaskTracker; these tasks run at the same time
mapred.tasktracker. reduce.tasks.maximun	1	The maximum number of Reduce tasks can be run on a TaskTracker; these tasks run at the same time
mapred.reduce. parallel.copies	30	Reduce startup more parallel copies for a large number of output map
io.sort.factor	100	More streams will be merged while sorting files
fs.default.name	hdfs://aiken:9000	The host IP and port of JobTracker
hadoop.tmp.dir	/root/data1	Hadoop default temporary path

**Table 3:** The results of *MK*-means.

	Cluster 1	Cluster 2	Cluster 3	Cluster 4	Cluster 5
20-20-hour precipitation (0.1 mm)	23	19	18	19	455
Average site pressure (0.1 hPa)	8364	9183	9966	6608	9876
Average wind speed (0.1 m/s)	23	20	20	22	20
Average temperature (0.1°C)	124	129	157	55	229
Average vapor pressure (0.1 hPa)	97	112	156	56	252
Average relative humidity (1%)	58	61	70	54	89
Sunshine hours (0.1 hour)	71	67	55	71	14
Minimum temperature (0.1°C)	72	76	118	−3	208
Maximum temperature (0.1°C)	188	193	206	129	265

**Table 4:** Throughput of the two different types of datasets.

File size	Block size	Test set	Write (MB/s)	Read (MB/s)
230 MB	64 MB	5	2.34	0.44
		6	5.17	10.34
